# Comparison of Different Post-Processing Algorithms for Dynamic Susceptibility Contrast Perfusion Imaging of Cerebral Gliomas

**DOI:** 10.2463/mrms.mp.2016-0036

**Published:** 2016-09-20

**Authors:** Kohsuke Kudo, Ikuko Uwano, Toshinori Hirai, Ryuji Murakami, Hideo Nakamura, Noriyuki Fujima, Fumio Yamashita, Jonathan Goodwin, Satomi Higuchi, Makoto Sasaki

**Affiliations:** 1 Division of Ultra-High Field MRI, Iwate Medical University, 2-1-1 Nishitokuta, Yahaba 028-3694, Japan; 2 Department of Diagnostic and Interventional Radiology, Hokkaido University Hospital, Sapporo, Japan; 3 Department of Radiology, Faculty of Medicine, University of Miyazaki, Miyazaki, Japan; 4 Department of Medical Imaging, Kumamoto University, Kumamoto, Japan; 5 Department of Neurosurgery, Kumamoto University, Kumamoto, Japan; 6 Laboratory of Biofunctional Imaging, Immunology Frontier Research Center, Osaka University, Suita, Japan

**Keywords:** dynamic susceptibility contrast, relative cerebral blood volume, magnetic resonance imaging, glioma

## Abstract

**Purpose:**

The purpose of the present study was to compare different software algorithms for processing DSC perfusion images of cerebral tumors with respect to i) the relative CBV (rCBV) calculated, ii) the cutoff value for discriminating low- and high-grade gliomas, and iii) the diagnostic performance for differentiating these tumors.

**Methods:**

Following approval of institutional review board, informed consent was obtained from all patients. Thirty-five patients with primary glioma (grade II, 9; grade III, 8; and grade IV, 18 patients) were included. DSC perfusion imaging was performed with 3-Tesla MRI scanner. CBV maps were generated by using 11 different algorithms of four commercially available software and one academic program. rCBV of each tumor compared to normal white matter was calculated by ROI measurements. Differences in rCBV value were compared between algorithms for each tumor grade. Receiver operator characteristics analysis was conducted for the evaluation of diagnostic performance of different algorithms for differentiating between different grades.

**Results:**

Several algorithms showed significant differences in rCBV, especially for grade IV tumors. When differentiating between low- (II) and high-grade (III/IV) tumors, the area under the ROC curve (Az) was similar (range 0.85–0.87), and there were no significant differences in Az between any pair of algorithms. In contrast, the optimal cutoff values varied between algorithms (range 4.18–6.53).

**Conclusions:**

rCBV values of tumor and cutoff values for discriminating low- and high-grade gliomas differed between software packages, suggesting that optimal software-specific cutoff values should be used for diagnosis of high-grade gliomas.

## Introduction

DSC perfusion imaging is widely used for brain tumors^1,2^ as well as acute stroke.^3,4^ It has several applications with respect to brain tumors, such as grading of primary gliomas,^1,2,5^ differentiation of tumor types,^6^ and discrimination of tumor recurrence from radiation necrosis.^7,8^ CBV, a parameter calculated from perfusion images, reflects microvascularity and is commonly used for glioma grading. It has been reported that a malignant (high-grade) glioma often shows increased vascularity and therefore higher CBV.^1,9,10^

Recently, different MR manufacturers, third-party workstation vendors, and academic groups have made available a variety of post-processing programs and algorithms for DSC imaging. However, these programs and algorithms substantially differ in terms of the maps and quantitative values in DSC as well as CT perfusion.^11–14^ In particular, there are several variations in the calculation method for CBV. CBV can be calculated as a ratio of the area under the curve (AUC) for tissues and that for large vessels as well as by deconvolution of the arterial input function (AIF). These differences in the calculation methods potentially affect the diagnostic performance of DSC perfusion imaging in different clinical applications. In addition, the same calculation method can sometimes yield varying results, probably because different software packages have different implementations even if they use the same basic algorithm. These differences between software packages have been reported to prevent standardization in perfusion imaging for stroke.^15^ The same may be true for tumor imaging; however, few reports have investigated differences in software used for processing DSC perfusion images of tumors.

The purpose of the present study was to compare different software packages for processing DSC perfusion images of cerebral tumors with respect to i) the relative CBV (rCBV) values calculated, ii) the cutoff value for discriminating low- and high-grade gliomas, and iii) the diagnostic performance for differentiating these tumors.

## Materials and Methods

### Subjects

This prospective study was conducted from May 8, 2009 to June 13, 2011. It was approved by the institutional review board, and informed consent was obtained from all the subjects. Thirty-six patients diagnosed with primary glioma (WHO grade II to IV) were included in this study. One patient for whom perfusion imaging was not conducted was excluded. Finally, 35 patients (17 men and 18 women) were selected to participate in this study. The mean age of the men and women was 59.8 (range 23–91) and 45.9 (range 8–74) years, respectively. Nine, eight, and 18 patients were diagnosed to have grade II, III, and IV gliomas based on histological specimens, respectively (Table 1).

### MR imaging

A 3-Tesla MRI (Magnetom Trio; Siemens, Erlangen, Germany) was used for DSC imaging. To minimize T1 effects of Gd leakage into the tumor tissue, half dose (0.05 mmol/kg) of Gd-DTPA was injected several minutes before DSC scanning, which was performed using the gradient echo (GRE)-EPI sequence. The scan parameters included TR of 1400 ms, TE of 32 ms, FOV of 230 mm, imaging matrix of 128 × 128, slice thickness of 5 mm, 19 sections, and 50 phases. Five seconds after starting the GRE-EPI sequence, the remaining half dose of the same contrast agent was injected at a rate of 3 mL/sec into the right antecubital vein, followed by saline chase of 20 mL at the same rate.

### Data analysis

The DSC data were post-processed and CBV maps were generated using 11 different algorithms of four commercially available software packages (GE Healthcare, Philips Medical Systems, Siemens Healthcare, and Infocom) and one academic program (Perfusion Mismatch Analyzer [PMA]) (Table 2).^11,12^ Of the 11 algorithms, six use the AUC method for CBV calculation, while five use deconvolution of the AIF. One of the six AUC algorithms uses baseline correction,^16^ and two implement curve fitting. All five deconvolution algorithms use singular value decomposition (SVD). The contralateral MCA was used as the AIF for deconvolution, and the same AIF position was used for all software packages.

ROI measurements were conducted on the CBV maps using the Dr.View/Linux software (Infocom Corporation, Tokyo, Japan) by a physicist (I.U., 4 years of experience in perfusion post-processing). Five ROIs (diameter of 2 mm) were manually placed in the high CBV area of the tumor (which shows the high CBV value on CBV map), and repeated for two to five sections depending on the size of the tumor. For example, if the tumor was large enough, five sections were maximum to measure, and if the tumor size was less than five sections, maximum numbers of sections which contain the tumor were used for the measurement (in this study the smallest tumor appeared in two sections). Ten ROIs of the same diameter were also placed in the contralateral, normal white matter. As these ROIs were used as the reference standard, not single ROI but 10 ROIs per section were measured to minimize the variations.

These ROIs were carefully placed to avoid large vessels (which appeared as a linear structure with high CBV), and the all the ROIs were copied to achieve exactly the same ROI positions for the different algorithms. The rCBV value of each tumor was then calculated as follows:

$$\text{rCBV}=\frac{\mathrm{average}{\mathrm{CBV}}_{\mathrm{tumor}}}{\mathrm{average}{\mathrm{CBV}}_{\mathrm{WM}}}$$

### Statistical analysis

Differences in the rCBV value between algorithms were compared for each glioma grade (grade II, III, and IV). Differences in rCBV value between glioma grades were also compared for each algorithm. These multiple comparisons were performed using Steel-Dwass non-parametric test.

For evaluating the diagnostic performance of rCBV, receiver operator characteristics (ROC) analysis was conducted for differentiation between grade II and III, grade III and IV, and low-grade (II) and high-grade (III and IV) gliomas. Optimal cutoff values for these discriminations were defined as the minimal distance from the ROC curve to the left upper corner. The area under the ROC curve (Az) values were compared between algorithms using the DeLong method with Bonferroni correction.^17^ Average Az values for the two calculation methods (AUC and deconvolution) were compared using Mann-Whitney’s *U*-test.

The statistical software package R (The R Project for Statistical Computing; http://www.r-project.org/) was used for all statistical tests, and *P* value less than 0.05 was considered as statistically significant for all tests.

## Results

### Comparison of rCBV values

**Fig 1.**
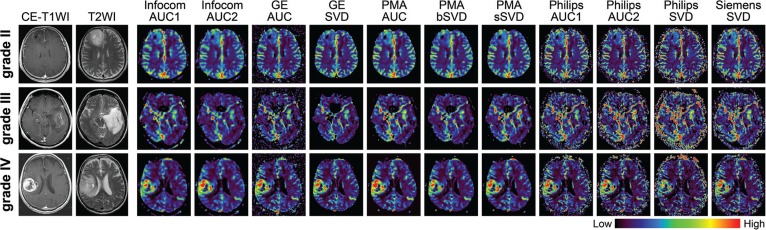
CBV maps generated by using all algorithms. Representative cases with grade II, III, and IV tumors are shown. All CBV maps are displayed with identical color bar, in which the window width is set to ten times of CBV in the normal white matter, and window level is set to half window width. Grade III and IV tumors have higher CBV than grade II tumor for all algorithms. The degree of CBV increase in the tumors (not only high grade, but also grade II tumors) and the amount of image noise differs between some software and algorithms. CE-T_1_WI, contrast enhanced T_1_ weighted images; T_2_WI, T_2_ weighted images; AUC, area under the curve; SVD, singular value decomposition

CBV maps were successfully obtained using all analysis algorithms (Fig. 1). After adjusting the window level and width for each map and using the same color scale, the overall CBV maps looked similar; however, the degree of CBV increase in the tumor (not only high grade, but also grade II tumors), conspicuity of large vessels, and image noise differed between algorithms.

**Fig 2.**
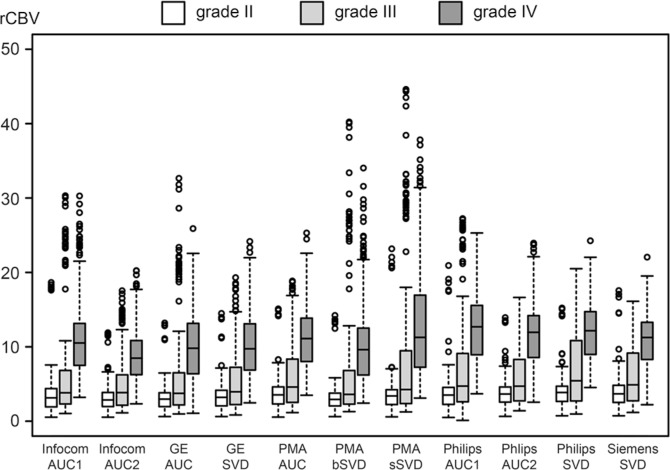
rCBV values using all algorithms for each tumor grade. rCBV increases with higher tumor grade for all algorithms. Significant differences are obtained between grade III and IV and between low-grade (II) and high-grade (III and IV) tumors by all algorithms. No significant differences are found between grade II and III. Note that the circles are outliers, which are defined by the distance greater than 1.5 times of interquartile range (between first and third quartile). AUC, area under the curve; SVD, singular value decomposition.

For all algorithms, the rCBV value increased with increasing tumor grade (Fig. 2). Significant differences in rCBV values were noted between grade III and IV and between low-grade (II) and high-grade (III and IV) tumors with all algorithms, while there were no significant differences between grade II and III tumors. As for the pairwise comparison between algorithms, a large number of pairs showed significant differences in rCBV especially for grade IV tumors (Table 3).

### Comparison of diagnostic performance

**Fig 3.**
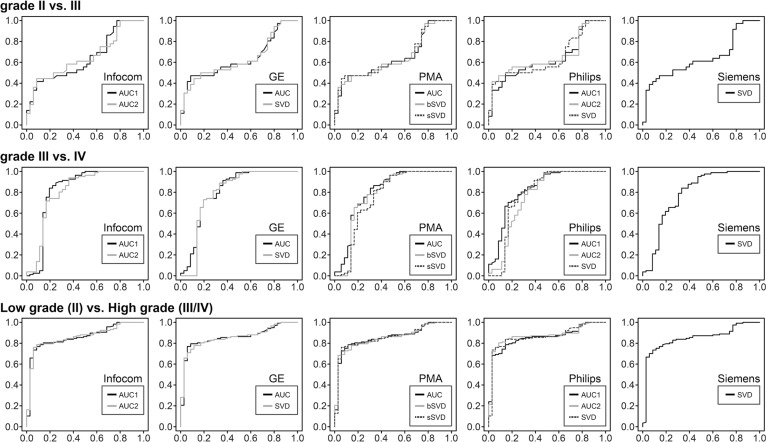
ROC curves for the differentiation of tumor grades. Curves for the different tumor grades appear similar for all algorithms. Variability of Az values differs substantially between tumor grades. Low Az variability is observed for the discrimination between grade II and III tumors and between low-grade (II) and high-grade (III/IV) tumors, whereas Az values are highly variable for discriminating between grade III and IV.

The ROC curves were shown in Fig. 3. The variability of Az values for comparison between grade II and III tumors were small (range 0.63–0.65), and there was no statistical difference between any pair of algorithms (Table 4). In contrast, variations in the optimal cutoff values were noted among algorithms (range 3.48–5.62).

Az values for the comparison between grade III and IV tumors showed greater variability (range 0.75–0.83) compared to the previous discrimination between grade II and III. Statistically significant differences were noted between PMA-AUC (0.81) and PMA-bSVD (0.78) (*P* = 0.044, Delong test). Optimal cutoff values also had considerable variability among algorithms (range 5.94–9.08).

For comparison between low- (II) and high-grade (III/IV) tumors, variability of Az values was small (range 0.85–0.87), and there were no statistical differences between any pair of algorithms. In contrast, the optimal cutoff values varied substantially among algorithms (range 4.18–6.53).

**Fig 4.**
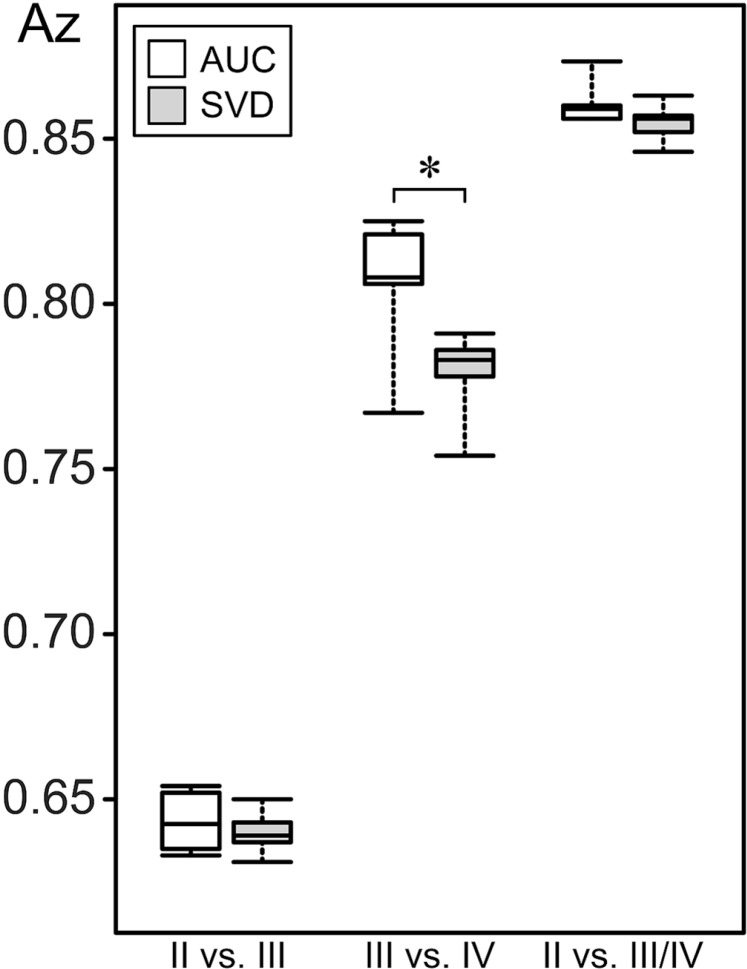
Average Az values of AUC and SVD algorithms for each tumor grade. Average Az values for AUC algorithms are slightly larger than for deconvolution. Significant differences are only observed in the comparison between grade III and IV tumors.

Overall, the average Az values were slightly larger for the AUC method than for the deconvolution. The difference was statistically significant only for the comparison between grade III and IV tumors (Fig. 4).

## Discussion

A previous study reported substantial differences in perfusion maps generated with different software using identical source data from patients with acute stroke.^12^ In that study, CBV maps of acute stroke were found to be less variable than CBF and MTT maps; however, only a few studies have examined variation in CBV values for cerebral tumors. In this study on patients with intracranial gliomas, we demonstrated that rCBV values differed depending on the software packages. Since the rCBV values were calculated relative to the values for the contralateral, normal white matter, the values were always normalized within each patient. Nonetheless, rCBV values differed considerably even when using the same software or different software using the same type of algorithm (such as AUC or SVD), which indicates that the detailed implementation of a specific analysis might vary among software. Tumor blood flow (TBF) and MTT were not analyzed in this study, as these are not generally used for the diagnosis of gliomas.

Compared to grade II and III, grade IV tumors had more variations in rCBV values across algorithms. Although smaller than high-grade tumors, grade II tumors also have slight variations. Conspicuity of large vessels and image noise were different among algorithms on visual assessment; therefore, the source of the difference might due to the difference in CBV value in highly perfused area. In addition, specific denoising procedures might differ between applications, and some software might have a ceiling threshold for CBV. Although the actual implementation of each perfusion analysis is often unknown, especially for commercial software, those differences in denoising steps possibly affect the sensitivity of smoothing effect in AUC and SVD, etc. In addition, the variations might be caused by the AIF determination in SVD, as CBV calculation is sensitive to AIF. This could be happened even in grade II tumors without contrast leakage.

Another possible source of variation among software may be baseline correction and curve fitting. Although pre-injection of Gd was used in this study, a baseline shift might have occurred due to contrast leakage during the first bolus. In fact, one software provided algorithms with and without baseline correction for contrast leakage (algorithm (1) and (2) in Table 2), and there was a significant difference in rCBV in only grade IV tumors between these two algorithms. As the high-grade tumors tend to have contrast leakage, baseline correction significantly affected the rCBV value in grade IV tumors in this study.

Although rCBV values varied across software, ROC analysis revealed that the diagnostic performance (Az values) for tumor grading did not differ significantly between software, although some differences were noted when discriminating between grade III and IV tumors. Previous studies have reported varying Az values for discrimination between low- (II) and high-grade (III and IV) tumors. For example, Hilario et al.^9^ reported an Az of 0.72 using a commercial software provided by an MR manufacturer, while Shin et al.^18^ reported an Az of 0.864 using an in-house software. To our knowledge, the highest Az of 0.97 was reported by Server et al.^19^ who used a third-party software. Our Az results (range 0.85–0.87) were intermediate. As overall differences between software packages were small in our study, the variations between previous reports are likely due to the patient population and glioma type, as the Az has been reported to be smaller (0.682) if only non-enhancing gliomas were studied.^20^

Further, we found that compared to the SVD method, the AUC method, which is simpler, had a significantly higher Az value for discriminating grade III and IV gliomas, although no significant differences were observed in discriminating grade II and III, and low-grade (II) and high-grade (III and IV) gliomas. The AUC method might have better diagnostic performance than SVD method, probably because the AUC method is simpler than SVD, or, SVD method might be susceptible to image noise and AIF determination, resulting in inferior diagnostic performance in glioma grading.

Although all software packages demonstrated similar diagnostic performances, the cutoff values for discrimination of different grades varied, probably because of the differences in rCBV values. Therefore, optimal cutoff values should be used for each software. Clinicians and academics should be careful when referring to cutoff values from other reports because different software might have been used for the analysis. In fact, cutoff values of previous reports often show considerable variability. For example, a cutoff values of 1.74 and 2.93 were reported for a commercial^9^ and in-house software, respectively.^18^

Again, in addition to the difference in the software, variations in tumor type may explain these differences in previous studies since rCBV of oligodendroglial tumors is higher than that of astrocytic tumors for low-grade tumors, and conversely, glioblastomas with an oligodendroglial component have lower rCBV than those without the component.^5,21^ Thus the ratio of astrocytic and oligodendroglial tumors might affect the cutoff values. Our results, which are based on identical source data, suggest that cutoff values varied substantially between software or algorithms. Our cutoff values (4.18–6.53) were higher than those reported previously, probably due to differences in software and methods for ROI measurement.

Another reason might be that we did not use vascular masking, which potentially increases rCBV values for high-grade tumors. Although we carefully positioned the ROI to exclude vascular pixels, tumor vessels might have been included in the ROI, increasing the cutoff value. In addition, CBV measurement critically depends on the number and size of ROIs. We averaged the maximum CBV for several smaller ROIs, usually more than 10 in each tumor. Therefore, the maximum CBV tended to be larger. We also pre-injected Gd to minimize T_1_ effects of contrast leakage. As most of the previous studies did not use pre-injection, CBV decreased in the enhancement area due to signal increase and decrease in estimated concentration of Gd.

This study has some limitations. First, the number of patients was relatively small. Previous studies reporting rCBV of cerebral glioma used higher number of patients. However, most of these studies were retrospective. Since we aimed to examine software differences, we conducted a prospective study to minimize sources of variation other than software differences, which limited the number of potential patients. Second, glial tumors with an oligodendroglial component should be discussed separately as rCBV values are different. However, this could not be achieved because of the small number of patients. Third, this study used ROI analysis, and not histogram analysis, which might have been superior to ROI analysis, as it decreases operator-dependent biases.^22^

## Conclusion

rCBV values of tumor and cutoff values for discriminating low- and high-grade gliomas differed between software packages, suggesting that optimal software-specific cutoff values should be used for diagnosis of high-grade gliomas.

## Figures and Tables

**Table 1.** Characteristics of patients

WHO grade	Pathology	Number of patients (male/female)	Age range
II	oligodendroglioma	6 (3/3)	18–62
	diffuse astrocytoma	3 (1/2)	23–46
III	anaplastic oligodendroglioma	6 (2/4)	33–54
	anaplastic astrocytoma	2 (0/2)	8–71
IV	glioblastoma multiforme	18 (11/7)	12–91

**Table 2.** List of software and algorithms

Manufacturer	Software	Analysis algorithm		AUC options	

				Baseline correction	Curve fitting
Infocom	Dr.View/Linux R2.5.0	(1)	AUC1 (type 0)	No	No
		(2)	AUC2 (type1)	Yes	No
GE	FuncTool 8.2.02	(3)	AUC (BrainStat GVF)	No	Yes
		(4)	SVD (BrainStat AIF)		
PMA	Ver. 3.4	(5)	AUC	No	No
		(6)	bSVD		
		(7)	sSVD		
Philips	R.2.6.1	(8)	AUC1 (model free)	No	No
		(9)	AUC2 (gamma)	No	Yes
		(10)	SVD (AIF)		
Siemens	VB11	(11)	SVD		

AIF, arterial input function; AUC, area under the curve; bSVD, block-circulant SVD; SVD, singular value decomposition; sSVD, standard SVD.

**Table 3.** *P* values of statistical comparison of rCBV between algorithms

WHO Grade	Algorithm									

	(2)	(3)	(4)	(5)	(6)	(7)	(8)	(9)	(10)	(11)
Grade II										
(1)	0.982	1.000	0.995	0.776	0.999	0.999	0.281	0.076	0.926	0.127
(2)	-	0.992	1.000	0.055	1.000	0.458	**0.003**	**<0.001**	0.135	**0.001**
(3)		-	0.999	0.486	1.000	0.982	0.090	**0.011**	0.698	**0.030**
(4)			-	0.101	1.000	0.593	**0.004**	**<0.001**	0.146	**0.001**
(5)				-	0.180	0.993	1.000	0.969	1.000	0.989
(6)					-	0.689	**0.008**	**<0.001**	0.210	**0.003**
(7)						-	0.822	0.341	1.000	0.583
(8)							-	1.000	0.996	1.000
(9)								-	0.832	1.000
(10)									-	0.946
(11)										-
Grade III										
(1)	0.990	1.000	0.975	0.920	1.000	0.839	0.744	0.172	0.864	0.524
(2)	-	1.000	1.000	0.151	0.998	0.236	0.093	**0.001**	0.177	**0.027**
(3)		-	1.000	0.522	1.000	0.488	0.377	**0.011**	0.347	0.156
(4)			-	0.348	0.996	0.268	0.194	**0.018**	0.172	0.096
(5)				-	0.788	1.000	1.000	0.752	1.000	1.000
(6)					-	0.786	0.549	0.093	0.825	0.341
(7)						-	1.000	0.975	1.000	1.000
(8)							-	0.889	1.000	1.000
(9)								-	0.996	0.991
(10)									-	1.000
(11)										-
Grade IV										
(1)	**<0.001**	0.490	0.478	0.861	0.204	0.464	0.052	**<0.001**	**<0.001**	0.961
(2)	-	**<0.001**	**0.002**	**<0.001**	**0.045**	**<0.001**	**<0.001**	**<0.001**	**<0.001**	**<0.001**
(3)		-	1.000	**0.006**	1.000	**0.001**	**<0.001**	**<0.001**	**<0.001**	**0.019**
(4)			-	**0.008**	1.000	**<0.001**	**<0.001**	**<0.001**	**<0.001**	**0.046**
(5)				-	**0.006**	0.957	0.955	0.083	**0.001**	1.000
(6)					-	**<0.001**	**<0.001**	**<0.001**	**<0.001**	**0.008**
(7)						-	1.000	0.992	0.728	0.815
(8)							-	0.846	**0.047**	0.505
(9)								-	0.926	**0.007**
(10)									-	**<0.001**
(11)										-

Bold type indicates statistical significance at 0.05 level (Steel-Dwass nonparametric multiple comparison test).

**Table 4.** Az and cutoff values for ROC analysis

Algorithm	II vs. III		III vs. IV		Low (II) vs. High (III/IV)	

	Az	Cutoff	Az	Cutoff	Az	Cutoff
(1)	0.64	4.70	0.82	7.17	0.86	5.05
(2)	0.65	3.48	0.81	6.58	0.86	4.77
(3)	0.63	4.80	0.81	7.29	0.86	4.80
(4)	0.63	4.05	0.79	6.00	0.86	4.40
(5)	0.64	5.62	0.81	6.95	0.86	5.62
(6)	0.64	4.18	0.78	5.94	0.85	4.18
(7)	0.65	5.42	0.75	6.69	0.86	5.30
(8)	0.63	4.46	0.83	8.79	0.86	5.20
(9)	0.65	4.65	0.77	8.27	0.87	6.53
(10)	0.64	4.84	0.79	9.08	0.86	4.84
(11)	0.64	4.63	0.78	7.14	0.85	5.66
